# A visual pollination mechanism of a new specialized pollinating weevil-plant reciprocity system

**DOI:** 10.3389/fpls.2024.1432263

**Published:** 2024-08-16

**Authors:** Jianjun Yue, Zhen Yan, Wei Liu, Ju Liu, Depo Yang

**Affiliations:** ^1^ School of Pharmaceutical Sciences, Sun Yat-Sen University, Guangzhou, China; ^2^ School of Traditional Dai-Thai Medicine, West Yunnan University of Applied Sciences, Jinghong, China; ^3^ Yunnan Key Laboratory of Southern Medicine Utilization, Institute of Medicinal Plant Development Yunnan Branch, Chinese Academy of Medical Sciences and Peking Union Medical College, Jinghong, China

**Keywords:** *Wurfbainia villosa*, pollinating weevil, ultraviolet pattern, gynandrium-like structure, visually mediated, mutualism

## Abstract

**Introduction:**

Pollinating flower-consuming mutualisms are considered exemplary models for studying coevolution due to their rarity. Visual cues are considered to have a major role in facilitating the evolution of floral patterns in these systems. We present a new specialized pollinating flower-consuming mutualism from the plant *Wurfbainia villosa*, which is a traditional Chinese herbal medicine, by a pollinating weevil, *Xenysmoderes* sp.

**Methods:**

In this study, We utilized monochrome plates for binary-choice tests to determine weevil color preferences, conducted behavioral choice experiments, using trackballs, photographed flowers and weevils, and employed blue sticky boards to attract weevils in the field.

**Results:**

Tests were conducted using colorpreferring weevils in both indoor and outdoor field systems, and validation experiments were performed. Behavioral tests were conducted to investigate the role of the visual cues in the pollinator attraction of *W. villosa*, which is a selfcompatible insect-pollinated plant that relies primarily on the *Xenysmoderes* sp. weevil for pollination due to its specialized gynandrium-like structure. Behavioral tests demonstrated that a blue color wavelength of 480 nm and the blue color system, as along with the UV-style pattern of the flowers, particularly the parts with specialized gynandrium-like structures in the labellum, were significantly attractive to both male and female weevils. These results were further confirmed through the field blue sticky board trap method.

**Discussion:**

These findings indicated that the interaction between *W. villosa* and *Xenysmoderes* sp. weevil was a novel symbiotic relationship involving pollinator flower consumption. Additionally, *Wurfbainia villosa* flowers developed specific visual cues of UV patterns and specialized structures that played a crucial role in attracting pollinators.

## Introduction

The evolutionary relationship between plants and insects has been a popular topic in evolutionary ecology research. Plants use direct or indirect defenses, such as releasing volatiles to attract pollinating insects (Dufa et al., 2004; Baldwin et al., 2006; Terry et al., 2007; Salzman et al., 2020) and natural enemies of herbivores (Rasmann and Turlings, 2007; Xin et al., 2012; Aartsma et al., 2017), and using neighboring plants for defense (Baldwin et al., 2006). The plant-pollinator mutualism is a classic model for studying adaptive evolution. Plants have diversified strategies for attracting pollinators. Insects use olfactory signals to locate host plants, and these signals are especially important for long-distance localization (Salzman et al., 2020). Pollinating insects often need to combine olfactory signals with visual cues at close range to locate hosts (Koski and Ashman, 2014). Weevils are among the oldest pollinating insects and are often used as a classic model for studying animal-plant evolutionary relationships (Salzman et al., 2020). These pollination systems are representative but relatively rare and have been found only in individual plant lineages, such as oil palm-*E. kamerunicus* weevils (Dhileepan, 1994; Yue et al., 2015; Haran et al., 2020), cycad-*R. furfuracea* weevils (Salzman et al., 2020), and orchid-orchid weevils (Nunes et al., 2018), among other exclusive pollination systems. The mechanism by which pollinating weevils locate host plants through specific mediating compounds has been identified through long-distance searches (Salzman et al., 2020). However, the mechanism by which pollinating weevils use visual cues to search for and locate host plant flowers in exclusive pollination systems remains unclear.

The investigation of the factors that contribute to the diversity of the flower colors has long been a subject of interest for botanists and evolutionary biologists. Various hypotheses have been proposed regarding the shifts in the floral color (Narbona et al., 2018). These hypotheses include non-adaptive evolution due to genetic drift (Wright, 1943) and the multidirectional effects of genes related to physiological or vegetative adaptations to environmental conditions (Rausher and Fry, 1993; Levin and Brack, 1995; Schemske and Bierzychudek, 2001; Warren and Mackenzie, 2001; Armbruster, 2002; Strauss and Whittall, 2006; Arista et al., 2013). Furthermore, the maintenance of flower color divergence may be attributed to the pleiotropic effects of the flower color genes on the herbivores and seed predators (Irwin et al., 2003; Carlson and Holsinger, 2010, Carlson and Holsinger, 2013). The evolution of flower color may also be influenced by a combination of genetic processes, biotic agents, and abiotic factors (Herrera, 1996; Galen, 1999a, Galen, 1999b; Ellis and Johnson, 2009; Ito et al., 2009; Schreiber et al., 2010; Steffan et al., 2024).

However, the role of selection pressure by pollinators is crucial in studies of adaptive evolutionary relationships between the plants and pollinators (Darwin, 1862; Schiestl and Johnson, 2013; Souto-Vilarós et al., 2018; Ramos and Schiestl, 2019). Pollinators are often considered important drivers of evolutionary shifts in plant flower color (Grant and Grant, 1965; Fenster et al., 2004; Harder and Johnson, 2009; Schiestl and Johnson, 2013; Gervasi and Schiestl, 2017; Johnson et al., 2020). Pollinators have a significant impact on the evolutionary adaptations of plants by transferring pollen between them (Waser and Price, 1981; Barrett and Harder, 1992; Waser et al., 1996; Schemske and Bradshaw, 1999). Pollinators exhibit convergent evolutionary selection for the flower color, especially in floras pollinated by the same pollinator or functional groups of similar pollinators (Fenster et al., 2004). Several studies have reported that animals that visit flowers have innate color preferences and can develop color preferences through associative conditioning (Lunau et al., 1996; Weiss, 1997; Pohl et al., 2008; Ings et al., 2009). Pollinator-mediated color preference selection behavior is a significant driver of the evolution of the floral pattern diversification (Kay and Sargent, 2009; van der Niet and Johnson, 2012).

Furthermore, pollinator selection and behavior can be influenced by visible light patterns (McCrea and Levy, 1983) and ultraviolet color patterns (Koski and Ashman, 2014; Peterson et al., 2015; Brock et al., 2016; Papiorek et al., 2016). For instance, certain flowers have developed ‘bull’s eye’ patterns to lure their pollinators (Manning, 1956; Free, 1970; Lunau et al., 1996; Johnson and Dafni, 1998; Dinkel and Lunau, 2001; Koski, 2020), which reduces the time taken for pollination by insects (Waser and Price, 1985; Leonard and Papaj, 2011; De Jager et al., 2017). Other flowers have developed human-visible and ultraviolet bull’s-eye patterns to attract pollinators. These patterns have been linked to the visual system of the pollinating insects (Asker, 1985; Briscoe and Chittka, 2001; Koski and Ashman, 2016; Koski, 2020). Assessing the relationship between the diversity of the plant flower patterns and the visual response of pollinators has been a popular topic in pollination ecology research in recent years. Currently, the focus is on Lepidoptera (Koski, 2020; Rodríguez-Castañeda et al., 2020), Hymenoptera (Koski, 2020; Rodríguez-Castañeda et al., 2020) and Diptera (Koski, 2020), as well as a few Coleoptera beetles (Johnson et al., 2020). These include pollen-feeding animals (Vernon and Gillespie, 1990; Gaum et al., 1994; Farnier et al., 2014). To date, studies on pollinator visual response mechanisms in pollinator weevil-plant mutualism are rare.

*Wurfbainia villosa* var. *villosa* (Lour.) Škorničk. & A. D. Poulsen (homotypic synonym: *Amomum villosum* Lour.) is a perennial semi-shade plant of *Amomum* Roxb. in the family Zingiberaceae (Yang et al., 2022; Chen et al., 2023). It has been cultivated for more than 1,300 years and is the most famous traditional Chinese medicine in China (Commission of Chinese Pharmacopoeia, 2020; Chen et al., 2023). *Wurfbainia villosa* is mainly found in the provinces of Yunnan, Guangdong, Guangxi, and Hainan in China and in Southeast Asian countries such as Laos and Thailand (Yue et al., 2021; Chen et al., 2023). It is a typical self-compatible insect-pollinated plant. *Wurfbainia villosa* has a specialized gynandrium-like structure, with the stigma positioned higher than the pollen sacs. Currently, key information on the ecology of insect-pollination in *W. villosa* remains unclear. In 2016, we conducted a study on the flowering characteristics of *W. villosa* and discovered a diverse range of flower colors. We hypothesized that a specific pollinating insect could be attracted to the color of the flowers of *W. villosa* and pollinate it. In 2017, we identified a small weevil that exclusively pollinated *W. villosa*. In 2024, a new strategy for pollination was validated using a specific part of the flower with a UV pattern that attracted a specific pollinating weevil.

A pollinating weevil (*Xenysmoderes* sp.) is a specialist pollinating insect of *W. villosa*. A reciprocal pollination system between this weevil and *W. villosa* has been identified. Our observations on the behavior of pollinating weevils of *W. villosa* revealed that diminutive (ca. 3 mm) weevils from the genus *Xenysmoderes* specialize in pollinating flowers. Based on our evidence that most pollination is performed by the pollinating weevils, these insects increase the initial fruit set of plants by 42%. We demonstrated that the pollinating weevil is the most efficient insect pollinator and mutualistic partner of *W. villosa* in Xishuangbanna Dai Autonomous Prefecture, Yunnan Province, China (Yan Z, unpublished data). Therefore, this system serves as a research model that is particularly suitable for investigating the effect of the flower color on the attractiveness of the pollinating weevil in an exclusively pollinating insect system. Specifically, we raise the following questions: (1) do *W. villosa* flowers have ultraviolet flower patterns? (2) do pollinating weevils prefer visual cues from the host plant flower color patterns? (3) Can UV patterns on specific parts of the host plant improve the ability of the pollinating weevil to select a location? (4) Does the pollinating weevil in this system use a visual pollination mechanism that relies on a proximity search to locate *W. villosa* through color preference?

## Materials and methods

### Test insects

The *Xenysmoderes* sp. weevils were collected from the Jinuo *Wurfbainia villosa* Planting Base (JN), Xishuangbanna Dai Autonomous Prefecture, Yunnan Province, China (GPS coordinates, 21°46’1’’N, 100°42’34’’E; Alt., 655 m). The site belongs to the tropical monsoon climate zone, with an average annual temperature of 22.9°C and an average annual rainfall of 1440 mm. The rainy season mainly occurs from May to September, and the planting mode is the natural understory. Dr. Chunyan Jiang (Institute of Zoology, Chinese Academy of Sciences) for identifying the weevils, *Xenysmoderes* sp. (**Supplementary Figure S1** and **Supplementary Movies S1**) and this weevil specimen is deposited in the laboratory of Prof. Yang Depo, School of Pharmaceutical Sciences, Sun Yat-Sen University. The adult weevils were placed in an insect rearing device and transferred to an artificial climate chamber with fresh *W. villosa* flowers. The device was kept under controlled conditions of 25 ± 1°C, a relative humidity (RH) of 75 ± 5%, and a photoperiod of light:dark (L:D) of 12 h:12 h. The reared adults were used as the source of the test insects. We collected approximately 19,562 adult weevils, which were reared in the laboratory for 1 week and then used in the experiment; a total of 18,540 adults were used in the experiment, and the rest continued to be reared for the establishment of an experimental population of this weevil.

### Color selection

Li et al. (2017) investigated the color tendency of *Bactrocera tau* using virtual wavelengths and value letters. They implemented a transformation between the RGB values of the colors and the virtual wavelengths with improvements. Thirteen colors were selected for the experiment, and the virtual wavelengths ranged from 400 nm to 640 nm (see **Supplementary Table S1**). White was used as the control. The RGB values of the wavelengths were entered into a computer system and printed on copper plate paper as test material.

### Experimental set-up design

The experimental setup consisted of a monochrome plate and a screen box. The screen box was 75 cm in length, 50 cm in width, and 50 cm in height. The screen had a mesh size of 0.0750 mm. A semicircular zipped opening with a radius of 15 cm was mounted on one side of the rectangle to serve as the insect release hole.

### Monochrome plate selection

Robust adult weevils were selected and acclimatized in a dark box for more than 2 h prior to being placed in the central position at the bottom of the experimental box. The control color (representing the control group) and the experimental color (representing the treatment group) were placed on the outside of the A-side and the B-side, respectively, with the remainder of the sides covered with black cardboard. The number of weevils on both side A and side B were counted 30 minutes after the insects were placed in the box. The experiments were conducted separately for females and males. Following each experiment, the inner wall and bottom of the experimental setup were wiped with 75% alcohol. The A-side and B-side of the experiment were separately changed in color and position, and the above experimental operations were repeated until all 13 colors were tested. The experiments were conducted in a separate laboratory room at a temperature of 25-28°C. Each release of 75-120 adult weevils was used as a replicate and repeated seven times for each color, with only one trial conducted per insect. The resulting data were recorded and analyzed separately using statistical methods. On the basis of personal observations, 30 minutes provided sufficient time for weevil activity to begin, and the results were not sensitive to this specific cutoff. For this reason, we chose to use the results from the 30-minute data.

### Behavioral choices of the weevil in response to different colors on a trackball insect behavior record

For the multicolor plate experimental setup, its experimental setup consisted of a cylindrical structure with openings at both ends. The outer ring of the cylinder was a rectangle 10.80 cm in width and 17.00 cm in height corresponding to the 10 different colors. Based on the 13 different colors in **Supplementary Table S1**, A total of 10 test colors (**Table 1**) were used, including a white control.

**Table 1:** Principal component scores of the pollinator weevil inspired by different colors on the trackball insect behavior recorder.

Test color	Virtual wavelengths (nm)	Lively factor score	Convergence factor score	Aggregate score
Light blue	420	2.53 ±0.06	0.43 ±0.15	1.83 ±0.08
Blue	440	1.09 ±0.52	0.56±0.24	0.88 ±0.42
Indigo violet	400	0.00±1.74	0.48±0.33	0.11±1.27
Sky blue	460	0.94±0.04	0.78±0.23	0.84±0.08
Orange	600	-1.30±0.89	-0.54±1.41	-1.02±0.95
Green	540	-2.55±0.58	0.02±0.96	-1.74 ±0.16
Yellow	580	-0.58±1.19	-0.63±0.70	-0.55 ±0.99
Indigo	420	0.09 ±0.83	0.23±0.95	0.11 ±0.80
Red	640	0.11±0.84	-0.44±1.43	-0.03±0.92
White	—	-0.33±3.86	-0.88 ±2.07	-0.44 ±2.11

An LC-300 trackball insect behavior recorder (OCKENFELS SYNTECH GMBH, Germany) was used to monitor behavioral parameters of the weevil; these included the crawling distance and speed. The trackball was configured with a 30 cm diameter white ball, and a color device consisting of 10 test colors was added. The experimental conditions were maintained at a temperature of 24-26°C and a relative humidity of 70%-75%. Prior to the commencement of the experiment, the insects were allowed to acclimatize to the dark box for more than 2 h. Thereafter, they were placed in the center of the trackball. The camera settings were then calibrated and adjusted, including exposure (-6), brightness (6), contrast (4), white balance, saturation, focus (auto), offset (5-40), add (0-4), and K-factor (0.2-0.4), and the light intensity and other main parameters were adjusted to successfully identify the motor behavior trajectory of each insect using the device. Then, the insect was placed in the center of the trackball and allowed to adapt for approximately 60 seconds before the crawling parameter data were recorded. The behavioral parameters of the insects were recorded every 300 seconds using a head-mounted trackball. The crawling behavior of the insects was also recorded using video, and their color preference was noted. The experiment was repeated six times for accuracy.

### Ultraviolet photographs of flowers and pollinating weevils

*Wurfbainia villosa* flowers and *Xenysmoderes* sp. adult weevils were collected from the base of JN. The flowers, including the labella, gynandrium-like structures, and pollen sacs, were photographed under UV light (UV lamp specification 40 W, Beijing Donglian Har Instrument Manufacturing Co., Ltd.). The control group consisted of photographs of the flowers under daylight conditions. The objective of the experiment was to elucidate the ultraviolet (UV) patterns of flowers and gynandrium-like structures, including the UV patterns of pollen carried by weevils.

### Behavioral selection of flowers by pollinating weevils under ultraviolet light

The experiment occurred in a screened experimental box measuring 75 cm in length, 50 cm in width, and 50 cm in height. The box was photographed under UV light. A number of robust adult weevils were selected and acclimatized in a dark box for more than 2 h prior to the experiment. They were then placed in the central position at the bottom of the experimental box (denoted by o), with the control flowers (paper model flowers were used as a control). The experimental group consisted of both *W. villosa* flowers (the treatment group), and a control group of paper model flowers placed at the left a-end (15 cm away from the o-point) and the right b-end (15 cm away from the o-point) at the bottom of the experimental box. The paper model flowers in the experiment were made by using flowers with the labellum of *W. villosa* removed, and its labellum was replaced with a fake labellum made of white paper. The number of weevils was counted at both the a-end and b-end 30 minutes after the insects were placed. Following each experiment, the inner wall and bottom of the experimental setup were wiped with 75% alcohol. The positions of the a-end and b-end were then exchanged, and the above experimental operations were repeated until the end of the experimental test. The test was conducted in a laboratory room at 25-28°C. In each replicate, 20-30 weevils were released, and the experiment was repeated 30 times. Only one test was allowed per insect, during which the flowers were replaced with fresh ones every 2 h. The experimental data were recorded and statistically analyzed.

### Blue sticky boards for the attraction of *Xenysmoderes* sp. weevils in the field

To test the attraction effect of the UV pattern of *W. villosa* flowers on field weevils, we conducted a series of experiments using blue-board sticky traps. These experiments were then extended to the natural breeding area of the weevil in Yunnan, China.

**Figure 1:**
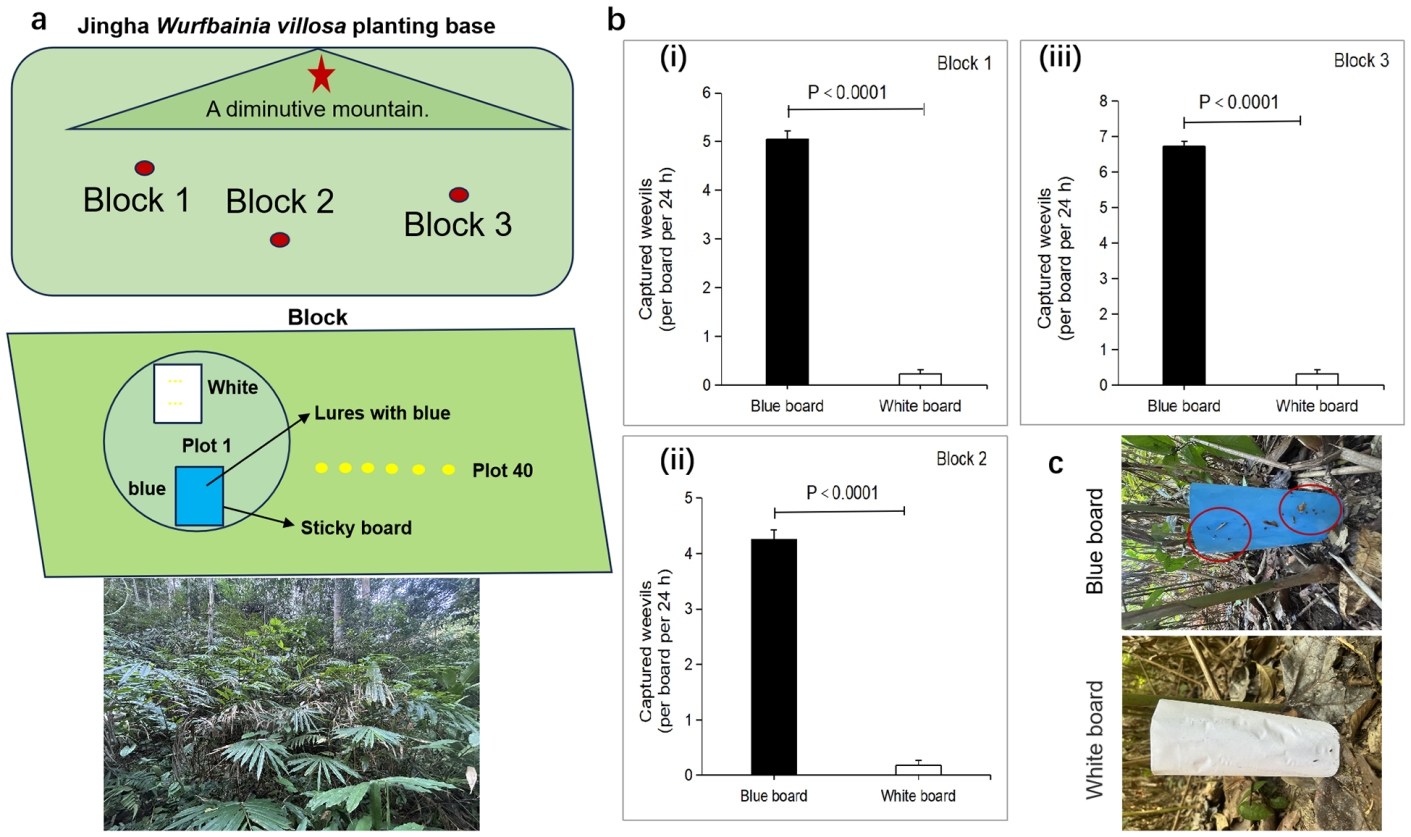
Blue sticky board attracts weevils to the planting base.**(A)** Schematic of the field trapping experiment at the Jingha *Wurfbainia villosa* planting base. **(B)** Number of captured weevils in the control (white sticky board) and blue sticky board groups in three blocks. **(C)** Average number of the captured weevils per board in the blue sticky board group (*n* =120 boards); this number was greater than that in the control group (*n* = 120 boards). All values are expressed as the mean ± SE. *P* values were determined by *t* tests.

The field experiment occurred at the Jingha *Wurfbainia villosa* plantation (JH) (21°89’77’’N, 100°87’87’’E, Alt., 582.6 m), which is located in the tropical monsoon climate zone. The rainy season is concentrated from May to September each year. The mean annual temperature is 22.9°C, and the mean annual rainfall is 1140.2 mm. *Wurfbainia villosa* was grown in a natural understory. The experimental design is shown in **Figure 1A**. Three plots were selected, and each had an area of no less than 2 ha and were spaced 30 m or more apart. Each plot was divided into 40 squares measuring 4×4 meters, with a 4-meter interval between each square. Two sticky boards were placed in each square: one blue sticky board measuring 25.0 cm in length and 18 cm in width and another white sticky board of the same dimensions; additionally, the specifications of the treatment group and the control group were the same. The sticky boards were erected vertically and placed 2.0 m apart. The same operation was carried out for 20 plots, with the other 2 plots set up in the same way as plot 1. The experiment was conducted in June 2023 at a temperature range of 26-32°C on a sunny day from 8:00 a.m. to 11:00 a.m. The number of weevils captured by the sticky boards was counted after 24 h.

### Statistical analysis

The preference of *Xenysmoderes* sp. weevils for the different color cues was assessed by calculating the convergence rate (CR), which was determined by the following formula:

CR = number of weevils responding in the different color palette areas/number of weevils supplied for testing

Analysis of variance (ANOVA) was employed to assess the rate of convergence in the color preferences of the *Xenysmoderes* sp. weevils for the different wavelengths. Prior to the application of ANOVA, the normality and heteroscedasticity of the variance were evaluated. Multiple comparisons of means were subsequently conducted via ANOVA. When the ANOVA results were statistically significant, multiple comparisons were made using Fisher’s protected least significant difference (LSD) test. The percentage data were transformed to a cosine square root prior to analysis. A *t* test was employed to assess the rate of convergence in color preference for the same wavelength between the weevil males and females, as well as for experiments involving comparisons between only two treatment groups. All data analyses were conducted using the statistical software SAS 9.1 (SAS Institute 1999), with the exception of the trackball insect behavioral recorder-multicolor selection tests; these tests were analyzed using the software SPASS18 to perform principal component analysis of convergent responses to color. Unless otherwise stated, the significance level for all tests was set at 5%.

## Results

### Behavioral responses of the pollinating weevils to different color plates

**Figure 2:**
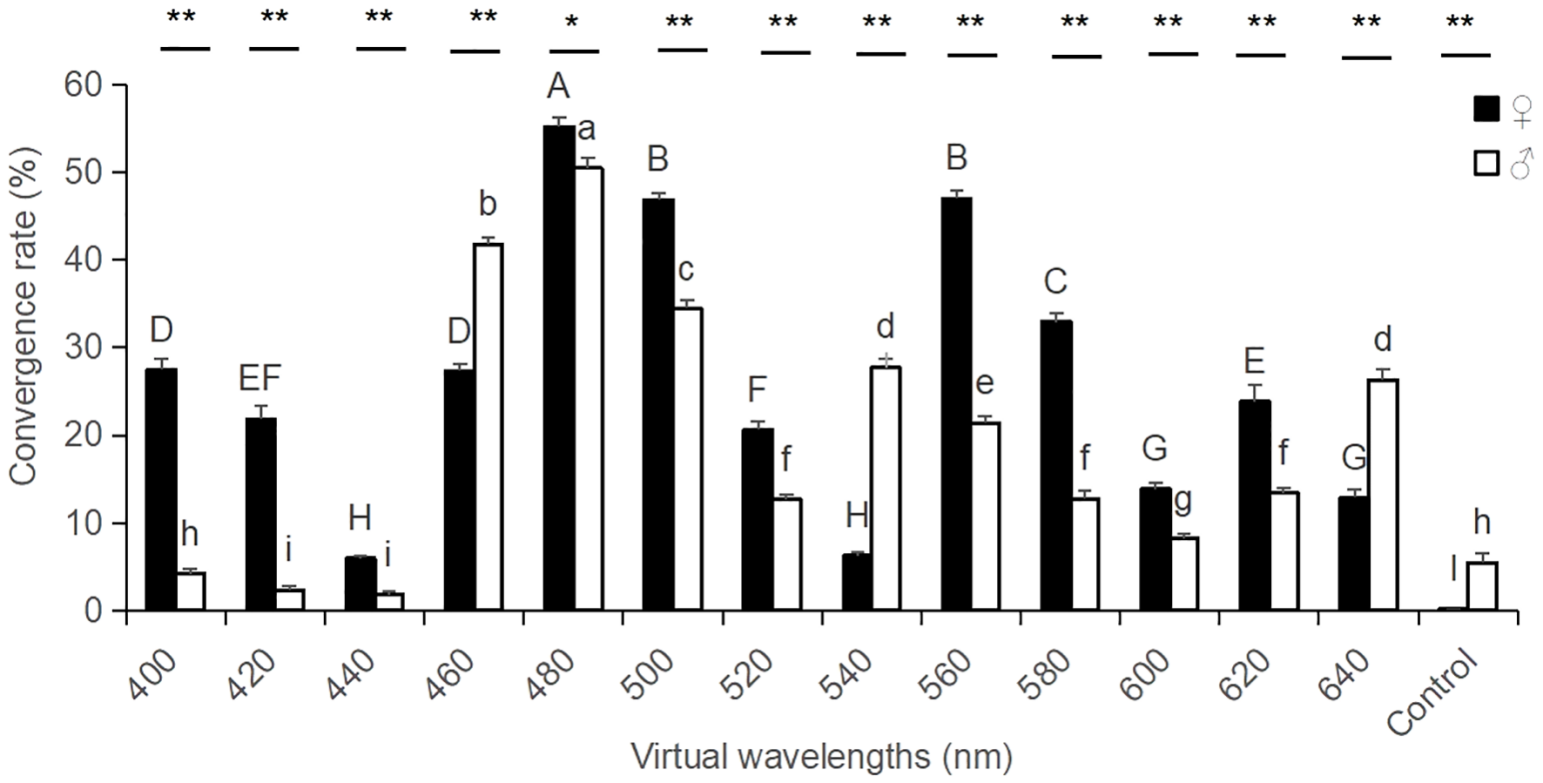
Convergence rates of male and female adult *Xenysmoderes* sp. weevils to monochromatic boards. All data are expressed as the mean ± SE. Mean values with different letters indicate significant differences between the color treatment groups (*P* < 0.05; Fisher’s LSD). * indicates a significant difference between the male and female treatment groups (*P* < 0.05; t test), and ** indicates a highly significant difference between the male and female treatment groups (*P* < 0.01; t test).

The results from the study demonstrated that for the monochromatic plate selection test, the convergence rate of the *Xenysmoderes* sp. weevil females exhibited a significant peak at 460-500 nm and a minor peak at 560-580 nm in their overall color preference across the different wavelengths. At 480 nm, the tendency rate of this weevil reached its maximum value (55.20%), and this value was significantly greater than those of the other treatment groups and the control group (*F*_1, 13_ = 331.64, *P* < 0.0001). At 560 nm, the tendency rate of this weevil reached its second largest value (47.02%), and this value was significantly greater than those of the other treatment groups and the control group, with the exception of a nonsignificant difference at 500 nm. At 400 nm, this weevil showed a certain preference (27.48%), which was significantly greater than those of the other treatment and control groups, with the exception of an insignificant difference at 460 nm (**Figure 2**). The convergence rate of the male weevil exhibited a primary peak in overall color preference across different wavelengths (460-500 nm), and this value was significantly greater than those of the other treatment and control groups (*F*_1, 13_ = 280.74, *P* < 0.0001). At 540 nm, the tendency rate of this weevil reached the next largest value (27.71%), which was significantly greater than those of the other treatment groups and the control group, with the exception that the difference at 640 nm was not significant (**Figure 2**).

The main peaks of the convergence rate of the weevil were more consistent for both females and males. Additionally, both sexes exhibited a similar color preference pattern, with a clear preference for the blue color at 480 nm (**Figure 2**). The wavelengths with the highest convergence rates were identical for both males and females. The convergence rate of this weevil reached its maximum at 480 nm, and this rate was significantly greater than those of the other treatment groups and the control group (female, *F*_1, 13_ = 331.64, *P* < 0.0001; male, *F*_1, 13_ = 280.74, *P* < 0.0001). Second, the 560-580 nm region of the green−yellow color system was the preferred wavelength for the weevil; moreover, the females exhibited a significant preference for 560 nm in yellowish-green color region, and the males exhibited a significant preference for 540 nm in the greenish color region. Also, some differences were observed between the two sexes. For the treatment groups with the same wavelength color, the convergence rates between both males and females were significantly different (except for 480 nm, *t* test, *P* < 0.05; for the other treatment groups, *t* test, *P* < 0.0001). With the exception of 460 nm, 540 nm and 640 nm, where the convergence rates of the males were significantly greater than those of the females, the convergence rates of the females were significantly greater than those of the males in the remaining treatment groups. These results indicated that the sensitivity to color differed between males and females.

### Behavioral choices of the pollinating weevils toward different colors as determined by the trackball insect behavior recorder

In this study, the convergent selection behavior of pollinating weevils were measured using ten different colors with a trackball insect behavior recorder. The following parameters were recorded: average speed, track length, vector length, straightness, upward length, and upward straightness. The results from the principal component analysis, using the maximum variance method for axis rotation, showed a Kaiser-Meyer-Olkin (KMO) value of 0.683. This result indicated that the sample capacity collected in this study was sufficiently large. The approximate chi-square value of Bartlett’s test of sphericity was 339.464, with a significance level of 0.000, indicating a high correlation between the original recorded parameters.

During the principal component extraction process, two principal components were extracted. These components accounted for 92.802% of the total variance. The first principal component, also known as the active factor, accounted for 68.417% of the total variance. The second principal component, known as the convergent factor, accounted for 24.385% of the total variance.

**Table 2** shows the component matrix, and the regression expressions between the active factor (y1), the convergence factor (y2), and the original crawling parameters are as follows:

**Table 2:** Component matrices for the principal component analysis of the crawling parameters of *Xenysmoderes* sp. weevil.

Test indicators	Ingredients	
	Active factor	Convergence factor
Vector length	0.951	-0.288
Average speed	0.928	-0.300
Track length	0.928	-0.300
Straightness	0.821	-0.163
Upward straightness	0.583	0.807
Upward length	0.682	0.723

y1 = 0.458Z_Average speed_+0.458Z_Track length_+0.470Z_Vector length_+0.405Z_Straightness_+0.337Z_Upward length_+0.288Z_Upward straightness_

y2=-0.248Z_Average speed_-0.248Z_Track length_-0.238Z_Vector length_-0.135Z_Straightness_+0.598Z_Upward length_+0.667Z_Upward straightness_

In the above equation, Z represents the normalized raw crawling parameters.

Our results indicated that the colors with the highest activity factor scores were light blue, blue, sky blue, red, indigo, indigo−violet, white, yellow, orange, and green. Similarly, the colors with the highest tendencies were sky blue, blue, indigo−violet, light blue, indigo, green, red, orange, yellow, and white. Regarding the composite score, the colors that rank highest in terms of the insect excitation composite score, in descending order, were light blue, blue, sky blue, indigo, indigo violet, red, white, yellow, orange, and green. Evidently, the colors with high insect-stimulating activity were determined by the activity factor score, the tropism factor score, and the composite score and were light blue, blue, and sky blue (**Table 1**).

In summary, the study results indicate that pollinating weevils have a preference for colors in the blue family. The most attractive colors for pollinating weevils were found to be light blue, blue, and sky blue (**Tables 1**, **2**). These findings were further validated through the use of the trackball insect behavior recorder method, as shown in **Figure 2**.

### Behavioral selection by the pollinator weevils on flowers of *W. villosa* under UV light

**Figure 3:**
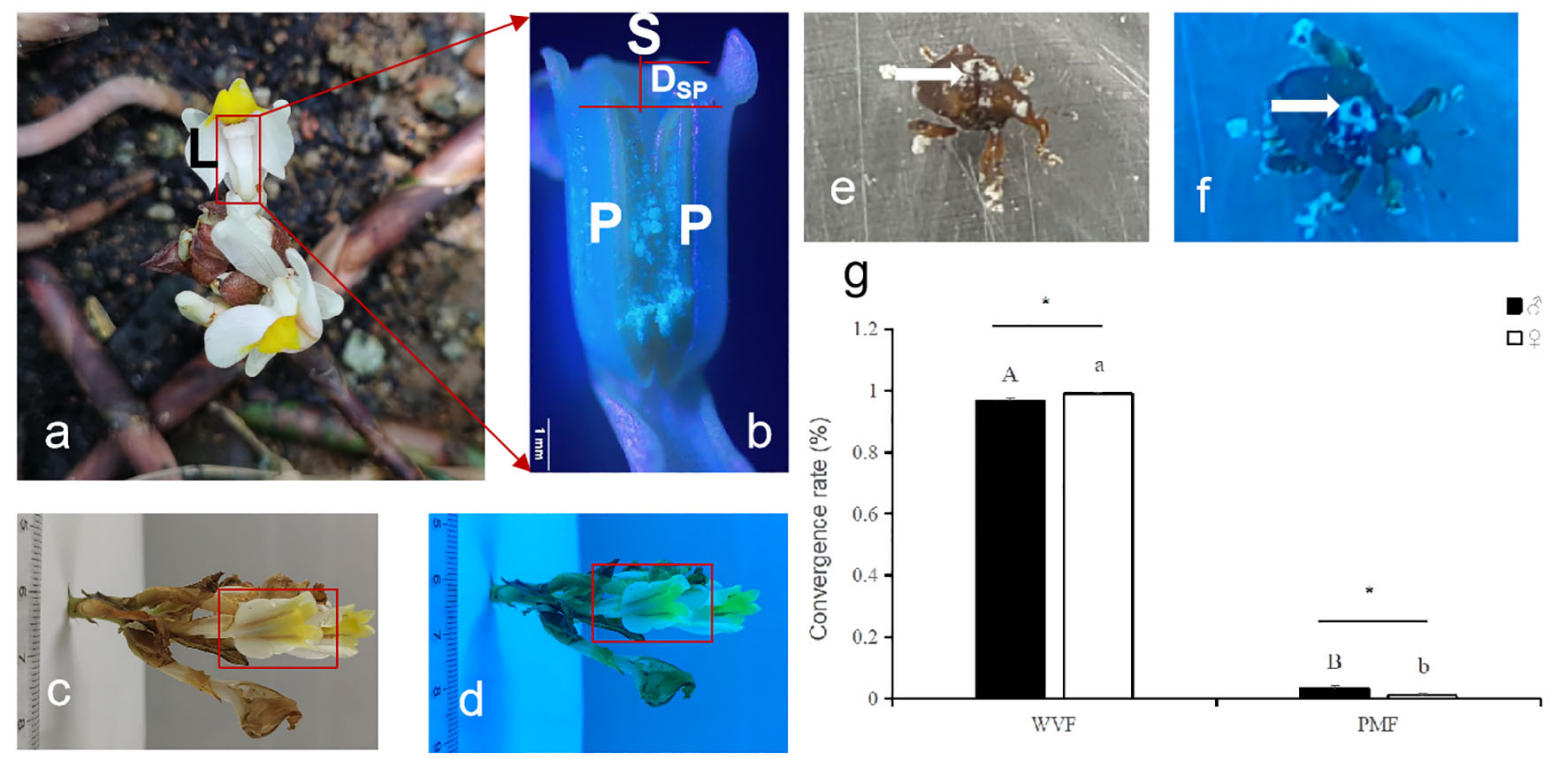
Flower color selection behavior of pollinators in a specialized pollinating mutualisms, *Wurfbainia villosa* – *Xenysmoderes* sp. weevil. **(A)**, *W. villosa* flower and its gynandrium-like structure (red box); L is the labellum. **(B)**, Diagrammatic representation of the gynandrium-like structure of a *W. villosa* flower, where S is the stigma, P is the pollen sac, and Dsp is the distance from the tip of the pollen sac to the stigma. **(C)**, Photograph of a *W. villosa* flower, along with the fuchsia double line on the flower’s labellum and the protruding and forked yellow−green ear-shaped structure at the tip of the labellum (red squares). **(D)**, Photograph of a sunburst sand flower under UV light, along with the blue-tinted double line on the flower’s labellum and the protruding and forked yellow−green ear-shaped structure at the tip of the labellum (red squares). **(E)**, Photograph of a sunlit *Xenysmoderes* sp. Weevil (white arrow indicates pollen). **(F)**, Photograph of a UV-lighted weevil (white arrow indicates pollen), and **(G)**, Convictive selection of the weevils on the flower and a control (pseudoflower) under UV light. All data are ± SEs. Different capital letters and lowercase letters indicate significant differences (*P* < 0.01, t test); * indicates significant differences (*P* < 0.05, t test).

The results of the study indicated that *W. villosa* has a unique gynandrium-like flower structure. The stigma protrudes from the middle of the two petal pollen sacs, and it is taller than the pollen sac. The stigma is connected to the pollen sacs and wrapped by a spatulate labellum. The labellum has a diverse color pattern and has a prominent and forked yellow−green ear-shaped structure at its tip (**Figures 3A, B**). Additionally, the *W. villosa* flower and its gynandrium-like structure, including the pollen of the pollen sacs, exhibited a bluish tinge under UV light compared to the control in daylight. Furthermore, the two purplish-red lines on the labellum also showed a bluish tinge under UV light (**Figures 3A–D**). Additionally, compared with those of the control, the adult *Xenysmoderes* sp. weevils, which have sheath wings and carry *W. villosa* pollen, exhibited overall bluish-purple coloration under UV light, regardless of sex (**Figures 3E, F**). The selection rate of the weevil group was significantly greater than that of the control group. The weevil showed a preference for the UV flower treatment group, regardless of sex (male, *F*_1, 58_ = 33.27, *P* < 0.0001; female, *F*_1, 58_ = 59.43, *P* < 0.0001; **Figure 3G**).

*W. villos*a exploited the UV pattern of its flowers and the diverse color patterns of its labellum to attract pollinators. The results from **Figure 2** and **Tables 1** , **2** were combined with those of **Figure 3** and **Tables 1** , **2** to verify that the preference of the weevil for the blue color family matched the UV pattern of the flowers and the color patterns of its labellum (**Figures 2**, **3**, **Tables 1**, **2**).

### Blue sticky boards for the attraction of *Xenysmoderes* sp. weevils in the field

The results of field trapping experiments showed that the blue sticky board had a strong attraction effect on *Xenysmoderes* sp. (**Figures 1A–C**). Compared with that in the white sticky board control group, the number of weevils captured in the blue sticky board treatment group was significantly greater than that in the control group within 24 h. The number of weevils captured in the treatment group was greater than 4 per sticky board, while that in the control group was less than 1 per sticky board (**Figure 1B**). For example, in block 3, the blue sticky board treatment group captured the greatest number of weevil adults in 24 h, and the number of weevil adults captured was 7 per sticky board (**Figure 1C**).

## Discussion

The results from our indoor and field experiments indicated that the *Xenysmoderes* sp. weevil exhibited a preference for the blue color scheme, and a significant preference for the 480 nm blue color was observed in both males and females. Second, the 560-580 nm region of the green−yellow color system was shown to be the preferred wavelength for the weevil, and females exhibited a significant preference for the yellowish-green color at 560 nm. Our new method involving the use of a trackball insect behavior recorder + swatch device further confirmed that the weevil also preferred the blue color scheme in its natural locomotion state. The UV pattern of the flower, particularly its unique gynandrium-like structure and labellum, was highly attractive to the weevil. Field experiments confirmed that the pollinating weevil showed a significant preference for blue plates. Therefore, our study clarified that the weevil has a visual preference for the blue color scheme and that the host plant *W. villosa* has UV patterns in its flowers and a specialized gynandrium-like structure, which significantly attracts the weevil. Thus, we discovered that the host plant *W. villosa* attracted pollinating weevils through the UV patterns in its specialized gynandrium-like structure in the *W. villosa* - *Xenysmoderes* sp. weevil specialized pollination system. Additionally, we revealed that pollinating weevils were drawn to the blue color schemes of flowers through the UV signal of the host plant. The visual pollination mechanism of the pollinating weevil was revealed through the UV signals emitted by the host plant’s flowers, aiding in proximity searching and localization. *Wurfbainia villosa* is a representative plant that is self-compatible and prone to insect pollination. Its flower pattern is unique and reflects the subtlety of the evolution of its pollination strategy. The specialized gynandrium-like structure is a key feature of this exclusively reciprocal pollination system. This model serves as a representative example and provides a new perspective for studying the adaptive evolution of the plant-pollinator reciprocal system.

In studies of adaptive evolutionary relationships between plants and pollinators, pollinator selection pressures have played an important role in evolution (Darwin, 1862; Schiestl and Johnson, 2013; Ramos and Schiestl, 2019). Several researchers have suggested that pollinator-mediated selection behavior for color preference is a key driver of floral pattern evolution (Grant and Grant, 1965; Lunau et al., 1996; Waser et al., 1996; Weiss, 1997; Schemske and Bradshaw, 1999; Fenster et al., 2004; Pohl et al., 2008; Ings et al., 2009; Kay and Sargent, 2009; van der Niet and Johnson, 2012; Gervasi and Schiestl, 2017; Johnson et al., 2020). Our findings showed that the pollinator *Xenysmoderes* sp. weevil preferred the blue lineage in an exclusive pollination system. This is the first report of the color preferences of an ancient pollinating beetle in such a rare, exclusive pollination system. The results from our study align with the tendency of generalized pollinating insects to favor flower UV-style patterns, as noted by Koski and Ashman (2014) and Peterson et al. (2015). Compound eyes are important visual sensory organs in insects whose main function is to discriminate between colors, shapes and other visual cues (Briscoe and Chittka, 2001), and their shape, size, color, number of auricles and photoreceptor cells can influence the size, acuity and sensitivity of an insect’s vision (Rutowski, 2000; Briscoe and Chittka, 2001; Xue et al., 2015). Therefore, we hypothesize that one of these factors must have influenced the color preference of this weevil. However, they contrast with the inclination of bees and birds toward yellow flowers, as observed by Papiorek et al. (2016). Different pollinator species exhibit similarities or differences in color preference, which may be related to biological factors such as the structure of their visual system (Briscoe and Chittka, 2001), flower structure and color patterns (McCrea and Levy, 1983;human-visible Asker, 1985; Lunau et al., 2009 ; Koski and Ashman, 2016; Koski, 2020), as well as the selection pressure of pollen-feeding animals (Vernon and Gillespie, 1990; Johnson et al., 2008; Farnier et al., 2014). Therefore, future studies investigating the evolutionary relationships between different pollinator systems and flower color patterns need to integrate the multifaceted effects of biological and other factors.

The findings from this study indicate that *W. villosa* flowers exhibit UV patterns, particularly at sites with special gynandrium-like structures. Additionally, the pollinating weevil displays a significant preference for the flower, particularly at the site of the gynandrium-like structure. The flowers attract the pollinator weevil through UV patterning and the evolution of specialized gynandrium-like structures. Host plant flowers have evolved pollinator-attractive colors or structures mediated by selection pressure for pollinator color preferences; for example, some flowers have evolved representative ‘Bull’s-eye’ patterns to attract their pollinators (Manning, 1956; Dinkel and Lunau, 2001; Lunau et al., 2009; Koski (2020), while some plants have evolved human-visible or UV bull’s-eye patterns to attract pollinators (Asker, 1985; Koski and Ashman, 2016; Koski, 2020). These patterns are related to the visual system of pollinators. Our results revealed a new structural and ecological function of the specialized gynandrium-like structure evolved by the flower. This structure attracts pollinators to the pollinator weevil, in addition to the reproductive function of its UV patterns, in a phallic *W. villosa*-weevil exclusive reciprocal pollination system. The diversity of the pollinator-mediated adaptive strategies for plant flower color patterning and structure is evident.

However, some flower-specific color signals can attract particular herbivores (Raguso, 2008). This can endanger the plant’s reproductive organs and reduce its reproductive success (Gómez, 2003; Irwin et al., 2003). This selective pressure from pollinators may have changed in intensity and direction due to herbivore feeding (Strauss, 1997; Hambäck, 2001; Herrera et al., 2002; Irwin et al., 2003; Irwin and Strauss, 2005; Agren, 2019; Ramos and Schiestl, 2019). The diversity of floral patterns evolves due to a balancing effect under the co-selective pressure of reciprocators and antagonists, as well as the variability in the evolution of the floral traits in host plants. This effect has been demonstrated by various studies (Fineblum and Rausher, 1995; Irwin et al., 2003; Caruso et al., 2010; Veiga et al., 2015; Ramos and Schiestl, 2019). Agren et al. (2013) and Vaidya et al. (2018) demonstrated the importance of pleiotropic influences from both biotic and abiotic factors in achieving a balanced strategy of evolutionary stability while maintaining evolutionary costs (Simms and Bucher, 1996; Strauss and Whittall, 2006).

Our study revealed that *W. villosa* flowers exhibited UV patterns, including a specialized gynandrium-like structure (**Figures 3A, B**), which attracted pollinating weevils. Additionally, the flowers had a variety of colors, including color patches, patterns, and veins on the labella. For example, the labellum has apical, middle, and basal color patches, with a prominent and forked yellow−green ear-shaped structure at its tip. In particular, the labellum has a high and striking yellow−green tip. The diversity of structures and colors in this labellum is determined by the curved ridge raised at the middle of the labellum, a pair of two nearly parallel lines extending across the entire labellum, and the two curved ridges appearing in a purplish-red color, as well as a blue tint in the UV pattern. The pollinating weevil is attracted to the diverse structure and color of the flower. The color pattern on the flower’s labellum helps the weevil detect the flower and then shows the correct direction to increase the chances of its successful pollination. This is due to a system of specialized pollinating insects. These findings are consistent with previous studies on the ecological roles of petal color patterns in attracting pollinators in other species (Manning, 1956; Lehrer et al., 1995; Hansen et al., 2012; Koski and Ashman, 2014). However, further systematic investigations and related assessment studies are needed to determine whether the flower color patterns of *W. villosa* can also discourage nectar predators and deter or attract floral predators.

The plants have diversified strategies for attracting pollinators. Pollinating insects use olfactory signals to locate host plants, and these signals are especially important for long-distance localization (Salzman et al., 2020). The results indicated that the visual pollination mechanism of the pollinating weevil was revealed through visual cues emitted by the host plant’s flowers, aiding in proximity searching and localization. The results of our study are similar to those reported by Koski and Ashman (2014). Pollinating insects often need to combine olfactory signals with visual cues at close range to locate hosts (Koski and Ashman, 2014). Weevils are among the oldest pollinating insects and are often used as a classic model for studying animal-plant evolutionary relationships (Salzman et al., 2020). These specific pollination systems are representative but relatively rare and have been found only in individual plant lineages (Dhileepan, 1994; Yue et al., 2015; Nunes et al., 2018; Haran et al., 2020; Salzman et al., 2020). The mechanism by which pollinating weevils locate host plants through specific mediating compounds has been identified through long-distance searches (Salzman et al., 2020). However, the mechanism by which pollinating weevils use visual cues and olfactory signals to cooperative search strategy for and locate host plant flowers in exclusive pollination systems remains one of the priorities for future research.

## Data Availability

The original contributions presented in the study are included in the article/**Supplementary Material**. Further inquiries can be directed to the corresponding authors.

## Supplementary material

Supplementary Table 1 List of colors and their virtual wavelengths and converted values.

Supplementary Figure 1 The flowers of *Wurfbainia villosa* are being pollinated by the weevils, *Xenysmoderes* sp.

Supplementary Movie S1 The flowers of *Wurfbainia villosa* are being pollinated by weevils. (the file are 7630 KB).
