# Supplementary material for: A visual pollination mechanism of a new specialized pollinating weevil-plant reciprocity system
Source: Front Plant Sci. 2024 Aug 16;15:1432263. doi: 10.3389/fpls.2024.1432263 (PMC11362035; doi:10.3389/fpls.2024.1432263)
Supplement: Supplementary Table 1 — List of colors and their virtual wavelengths and converted values. [file Table1.docx]

**Table S1.** List of colours and their virtual wavelengths and converted values.

| Virtual wavelengths (nm) | Colour | RGB values | *L***a***b** values | CMYK values (%) |
| --- | --- | --- | --- | --- |
| 400 |  | 110, 0, 166 | 27.22, 30.28, 0.91 | 61, 91, 64, 28 |
| 420 |  | 85, 0, 255 | 29.34, 19.66, 39.22 | 56, 79, 100, 36 |
| 440 |  | 0, 0, 255 | 28.48, 7.24, 46.03 | 65, 70, 100, 39 |
| 460 |  | 0, 102, 255 | 48.80, 4.12, 38.91 | 54, 56, 96, 7 |
| 480 |  | 0, 204, 255 | 71.03, -19.66, -22.79 | 60, 14, 15, 0 |
| 500 |  | 0, 255, 128 | 69.23, -24.19, 29.38 | 53, 17, 65, 0 |
| 520 |  | 36, 255, 0 | 65.36, -22.21, 53.72 | 54, 24, 95, 0 |
| 540 |  | 109, 255, 0 | 70.36, -14.53, 62.49 | 43, 24, 93, 0 |
| 560 |  | 182, 255, 0 | 74.32, -7.35, 73.24 | 32, 25, 96, 0 |
| 580 |  | 255, 255, 0 | 82.23, -0.06, 77.27 | 16, 22, 87, 0 |
| 600 |  | 255, 177, 0 | 71.88, 16.15, 64.19 | 16, 42, 85, 0 |
| 620 |  | 255, 98, 0 | 58.99, 34.96, 55.61 | 21, 65, 90, 0 |
| 640 |  | 255, 20, 0 | 44.90, 53.20, 40.56 | 32, 91, 93, 1 |
