# Supplementary figures and images for: A visual pollination mechanism of a new specialized pollinating weevil-plant reciprocity system
Source: Front Plant Sci. 2024 Aug 16;15:1432263. doi: 10.3389/fpls.2024.1432263 (PMC11362035; doi:10.3389/fpls.2024.1432263)

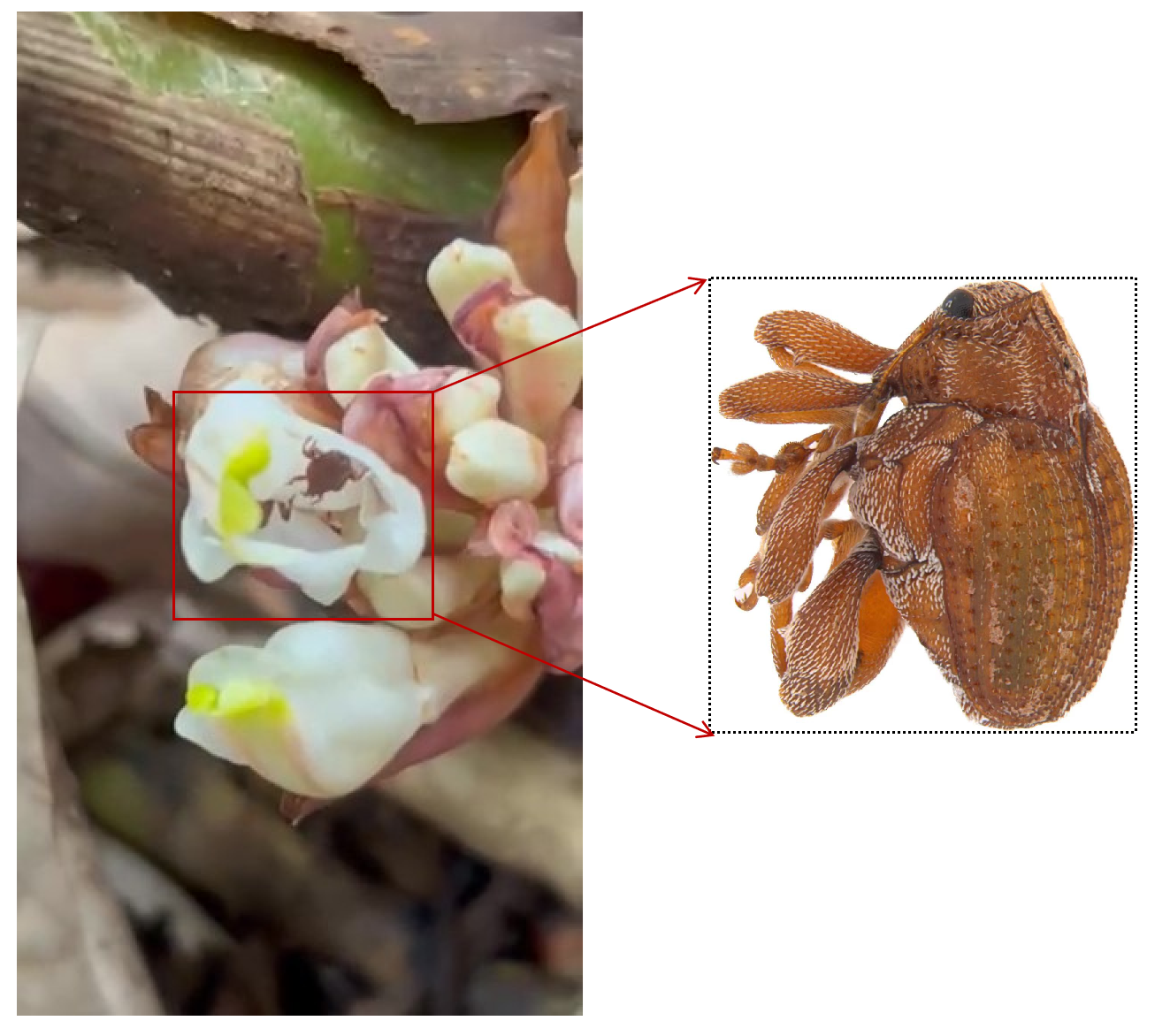

Supplement: Supplementary Figure 1 — The flowers of Wurfbainia villosa are being pollinated by the weevils, Xenysmoderes sp. [file Image1.tif]
